# Hydrodissection as a Novel Alternative After Failed Management of a Cervical Pregnancy With Methotrexate: Case Report and Literature Review

**DOI:** 10.7759/cureus.52556

**Published:** 2024-01-19

**Authors:** Natalia Cárdenas-Suárez, Paulette Urrutia-Villamil, Taymara Reyes-Jimenez, Olga Pereira-Diaz

**Affiliations:** 1 Department of Obstetrics and Gynecology, University of Puerto Rico Medical Sciences Campus, San Juan, PRI; 2 Department of Obstetrics and Gynecology, San Juan City Hospital, San Juan, PRI

**Keywords:** cervical ectopic pregnancy, ectopic pregnancy, methotrexate, hydrodissection, cervical pregnancy

## Abstract

Cervical ectopic pregnancies (CEPs) are rare and life-threatening diagnoses. Risk factors have been associated with CEPs, yet their etiology and pathogenesis remain unknown. Timely intervention is vital for successful outcomes, yet it is challenged as there is no standardized approach to treatment. We present the case of a 42-year-old woman diagnosed with CEP following five weeks and one day of amenorrhea. The patient was treated with a two-dose regimen of intramuscular methotrexate (MTX) but failed to respond. Ultrasound-guided intrasac MTX injection was considered a secondary treatment. However, spontaneous expulsion was observed after administering lidocaine at different cervical points. Hydrodissection following systemic MTX could present a novel alternative for treating CEP. Expulsion of pregnancy after hydrodissection could be associated with tissue necrosis and/or destabilized implantation of pregnancy, secondary to the effects of MTX. Further research is vital for evaluating the underlying mechanisms for expulsion and the role of hydrodissection following MTX in treating CEP.

## Introduction

Cervical ectopic pregnancies (CEPs) are rare, accounting for less than 1% of all pregnancies [1, 2]. This subtype of ectopic pregnancy results from abnormal embryo implantation within the cervix [1, 2]. Due to the high vascularity and limited space within the cervix, CEPs are associated with high morbidity and mortality due to the risk of hemorrhage [1, 2]. Early diagnosis of this life-threatening condition is vital and warrants timely intervention. The etiology and pathogenesis of CEPs remain unknown. However, identifying risk factors could aid in targeting prevention and treatment strategies.

The diagnosis of a CEP is based on clinical presentation and ultrasound (US) findings. The most common symptom is painless vaginal bleeding. As for US findings, criteria for diagnosis include the absence of an intrauterine pregnancy, a gestational sac below the level of the internal cervical os, the absence of a “sliding organ sign,” and vascularity around the gestational sac detected by Doppler [2].

Several alternatives have been proposed for the management of CEPs. However, no consensus has been established. Conservative measures have become the standard approach for clinically stable women who desire fertility preservation [3]. Surgical approaches are often reserved for hemodynamically unstable patients or those who fail to respond to conservative measures [1].

Methotrexate (MTX) has been widely used as a conservative treatment for CEP [1]. Nevertheless, its success rate may vary depending on clinical findings, dosing regimen, and administration method. We report the case of a CEP unresponsive to MTX yet resolved by spontaneous expulsion following cervical hydrodissection.

## Case presentation

A 42-year-old female, G2P2012, presented to the obstetrics and gynecology emergency room complaining of painless vaginal bleeding following five weeks and one day of amenorrhea. She had no systemic illness. Her obstetric history was remarkable for two at-term births, each delivered by cesarean section, and a ruptured tubal ectopic pregnancy, treated with exploratory laparotomy. The patient also denied having drug allergies or engaging in toxic habits, such as smoking.

**Figure 1:**
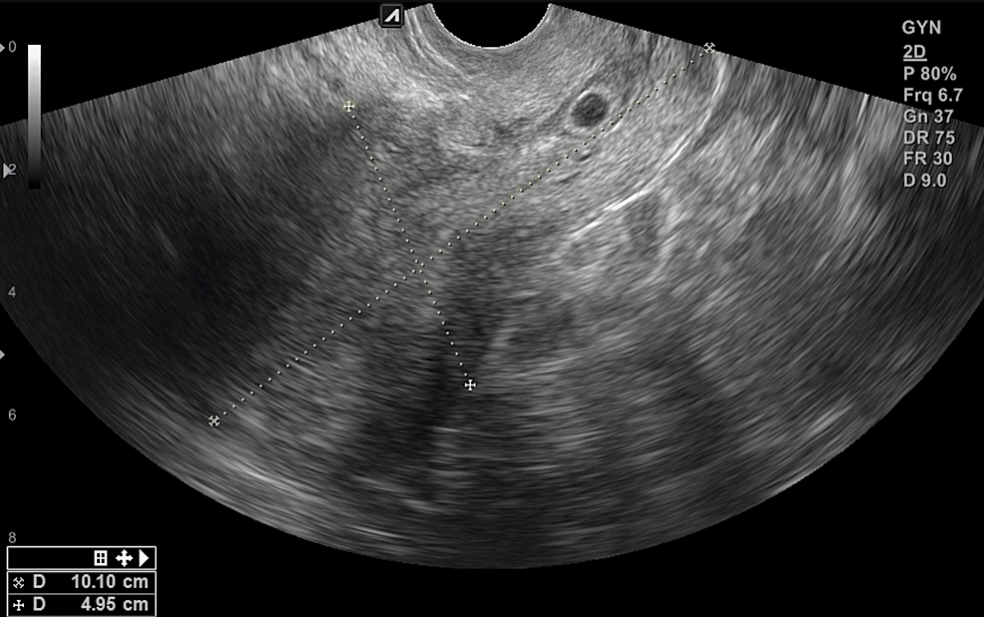
Transvaginal ultrasound performed at the initial evaluation shows an anechoic structure with a poorly delimited internal ring observed within the posterior lip of the cervix.

Upon arrival, the patient was hemodynamically stable. A pelvic exam revealed scant blood within the vaginal vault but no active bleeding from the cervical os. Transvaginal US (TVUS) showed an anteverted, retroflexed uterus with trilaminar endometrium and an empty uterine cavity. An anechoic, oval-shaped structure of 0.71 cm with an internal, poorly delimited ring and peripheral hyperechoic tissue was observed within the posterior lip of the cervix (Figure 1).

Evaluation of the fundus was limited by refractory shadows exerted by myomas within the anterior wall and lower uterine segment. These measured approximately 4 cm in their greatest diameter. As for the adnexa, only the left ovary was observed and was remarkable for a 2.1 cm x 2.0 cm cystic structure within the parenchyma. The beta-human chorionic gonadotropin (beta-hCG) test resulted in 3,950 mIU/mL.

The patient was discharged and instructed to return within 48 hours for reevaluation. Upon reassessment, TVUS evidenced pregnancy progression. The oval-shaped structure within the cervix grew to 1.05 cm, and its well-delimited internal ring measured 0.26 cm. Evaluation with a Doppler showed bidirectional flow surrounding the cervical structure. Βeta-hCG also increased to 6,018 mIU/mL. The patient was informed about her diagnosis and counseled regarding treatment. She agreed to conservative management for fertility preservation. Thus, systemic MTX was administered as a two-dose regimen (50 mg intramuscular MTX on days one and four). Rho(D) immune globulin (RhIG) was placed as the RhoGAM vaccine given her O-negative blood type.

The pregnancy continued to progress. The TVUS showed a 1.19 cm gestational sac and a 0.49 cm yolk sac. The development of a fetal pole with a crown-rump length of 0.27 cm, without cardiac activity, was observed. Βeta-hCG further increased to 9,154 mIU/mL (Table 1).

**Table 1:** Progression of cervical pregnancy from day one to day nine wGA: weeks gestational age; GS: gestational sac; YS: yolk sac; CRL: crown-rump length; IM: intramuscular; OR: operating room

	Day 1	Day 3	Day 7	Day 9
Gestational age	5 1/7 wGA	5 3/7 wGA	5 6/7 wGA	6 1/7 wGA
Beta-hCG	3950 mIU/mL	6018 mIU/mL	5214 mIU/mL	9154 mIU/mL
Sonographic findings	GS: 0.71 cm with decidual reaction. Located at the endocervix.	GS: 1.05 cm, YS: 0.26 cm. Located at the endocervix.	GS: 1.12 cm, YS: 0.41 cm, CRL: 0.16 cm. Located at the endocervix, posterior cervical lip.	GS: 1.19 cm, YS: 0.49 cm, CRL: 0.27 cm. Located at the endocervix, posterior cervical lip.
Intervention	None	Methotrexate 50 mg IM	Methotrexate 50 mg IM	Admitted to the OR

Two high-risk pregnancy specialists were consulted, and a consensus was reached for a US-guided MTX intrasac injection with Foley catheter balloon tamponade in case of hemorrhage.

**Figure 2:**
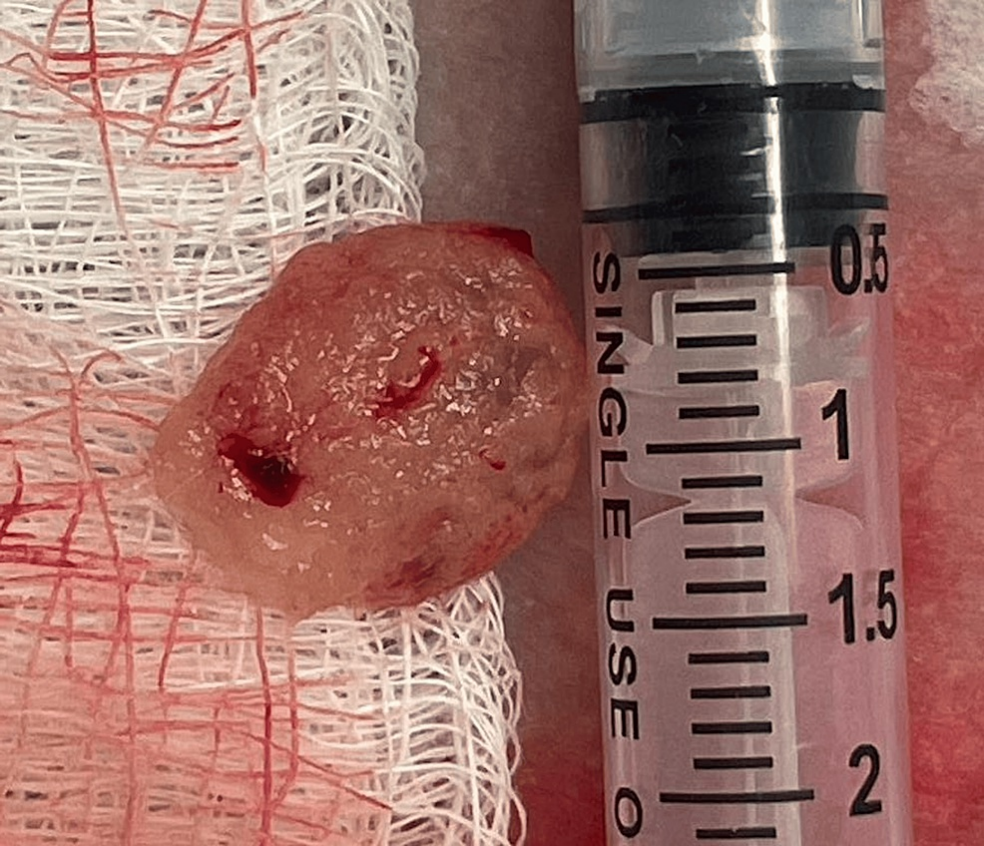
Expelled tissue after administration of lidocaine at 4 o’clock and 8 o’clock.

The patient was oriented about the findings, MTX failure, and consensus. After agreeing with the new management protocol, the patient was admitted and taken to the operating room, where she was placed in a lithotomy position, cleansed, and draped under sterile conditions. The TVUS confirmed the CEP remained in place. Aided by a sterile speculum, the cervix was visualized and cleaned with Betadine solution. A tenaculum was placed at 12 o'clock, then the cervix was infiltrated with 5 mL of 1% lidocaine at 4 o’clock and 8 o’clock with a 20G needle and syringe. Promptly, spontaneous expulsion of CEP was observed (Figure 2).

The TVUS confirmed CEP expulsion, as no sac was identified within the canal. As heterogeneous tissue was observed near the internal os, 50 mg of MTX was administered within the area with a 26G puncture needle under US guidance. No bleeding was observed after the infiltration. The estimated blood loss was 5 mL. The pathology report further confirmed CEP expulsion of pregnancy.

The patient had an uneventful recovery and was discharged two days after her procedure. The patient's beta-hCG values were followed until non-pregnant levels were reached (Table 2).

**Table 2:** Declining beta-hCG values after spontaneous expulsion of cervical pregnancy * Spontaneous expulsion of pregnancy occurred on day nine. beta-hCG: beta-human chorionic gonadotropin

	Day 9*	Day 11	Day 13	Day 17	Day 21
Beta-hCG	9154 mIU/mL	759 mIU/mL	260 mIU/mL	37 mIU/mL	3 mIU/mL

## Discussion

Cervical ectopic pregnancies are diagnosed based on symptoms and TVUS findings. In this case, the clinical presentation was initially equivocal. The diagnosis was based on pregnancy progression after 48 hours. This period allowed gestational sac growth, yolk sac development, enhancement of surrounding vascularity, and an increase in beta-hCG.

The etiology and pathogenesis of CEPs are currently unknown. The occurrence of CEP has been associated with damage to the uterine cavity and interference with pregnancy implantation [2]. Potential risk factors include in-vitro fertilization, pelvic infection, myomas, post-surgical trauma due to cesarean section or uterine curettage, a history of abortions, and the use of intrauterine contraceptive devices [3]. Myomas and post-surgical trauma were identified as potential risk factors in this case. Myomas within the anterior wall and lower uterine segment could have interfered with muscle contractility, impeded embryo transport, and obstructed implantation by compression of the endometrium [4]. Myomas are also known to alter the gene expression of basic fibroblast growth factor (bFGF), platelet-derived growth factor (PDGF), and homeobox A10 (HOXA10). Thus, the increased bFGF and PDGF could have altered angiogenesis, while the decreased expression of HOXA10 could have reduced endometrial receptivity within the region [4]. The patient's history of pelvic surgeries was another potential risk factor for this case. Fibrotic tissue resulting from previous cesarean sections could have further promoted unfavorable conditions for implantation due to reduced vascularity and anatomical distortion within the lower uterine segment [5].

Approximately 50% of women who experience ectopic pregnancies lack identifiable risk factors [1]. The American College of Obstetrics and Gynecology (ACOG) establishes that women with a history of ectopic pregnancy are at increased risk of recurrence [1]. The extent of the increase depends on other factors such as the number of prior ectopic pregnancies, history of pelvic surgeries, use of assisted reproductive technology, older age, smoking, and infertility [1]. The patient, in this case, had at least a 10% increased risk of recurrence, given her prior ectopic pregnancy. However, her risk could have been even higher, as ACOG’s estimated risk does not account for prior pelvic surgeries or older age. It is also vital to recall that ACOG’s estimated risk does not discriminate among the types of ectopic pregnancies. Multiple theories have been proposed for explaining recurrences. Nevertheless, most address mechanisms involving improper tubal function, which may not necessarily apply to CEP. As these lie distal to fallopian tubes, additional unidentified factors may be responsible for CEPs presenting as recurrences.

There is currently no standardized approach for managing CEPs. Methotrexate is the recommended first-line treatment for hemodynamically stable women, administered systemically or locally with US guidance [1]. Based on clinical criteria, intramuscular MTX may be administered as a single-, two-, or multi-dose regimen. The success rate for each regimen has been documented as a varied range of 70%-90% for the single-dose, 80%-90% for the two-dose, and 89%-96% for the multi-dose regimen [3, 6]. Another alternative is the US-guided MTX intrasac injection, with or without the need for concomitant procedures for bleeding control or residual tissue removal. The success rate of MTX injection is yet to be established. Nevertheless, research has evidenced that combining systemic and intrasac injections of MTX increases the chance of successful outcomes [2].

Various measures have been proposed for predicting the risk of MTX failure. These include a gestational age >9 weeks, beta-hCG levels >10,000 mIU/mL, crown-rump length >10 mm, and the presence of cardiac activity [7]. All predictive measures were below their corresponding threshold in this case. Thus, a two-dose intramuscular MTX regimen was administered for higher chances of success than single-dose regimens but minimizing the potential side effects reported with multidose protocols. However, the CEP failed to respond.

Spontaneous expulsion was observed following lidocaine administration within the cervix, suggesting hydrodissection as a potential alternative for management. Hydrodissection uses pressurized fluid to displace one tissue from another, entering the plane of least resistance. This technique has been used as an aid in the treatment of tubal, interstitial, and abdominal pregnancies with successful results [8, 9]. Various methods have been reported, depending on the location of the pregnancy and proximity to tissues. However, no study has reported the use of hydrodissection for treating CEPs.

Given our findings, we propose cervical hydrodissection as a novel alternative after failed management of CEPs with MTX. For this, US-guided, normal-pressure hydrodissection may be performed with a 20G needle and a syringe containing 5-10 mL of a vasoconstricting solution (e.g., lidocaine 1%). First, confirm the CEP location and needle positioning at a 20-30 mm depth under US guidance. Then, infiltrate the solution at single or multiple sites within the implantation site until the pregnancy is displaced. Although other solutions may be used (e.g., normal saline or 5% dextrose in water), we encourage using vasoconstricting agents, as these can potentially decrease the risk of hemorrhage involved with CEPs and impede its progression by interrupting blood flow. Direct observation of tissues under US guidance further increases the accuracy and effectiveness of the hydrodissection.

**Figure 3:**
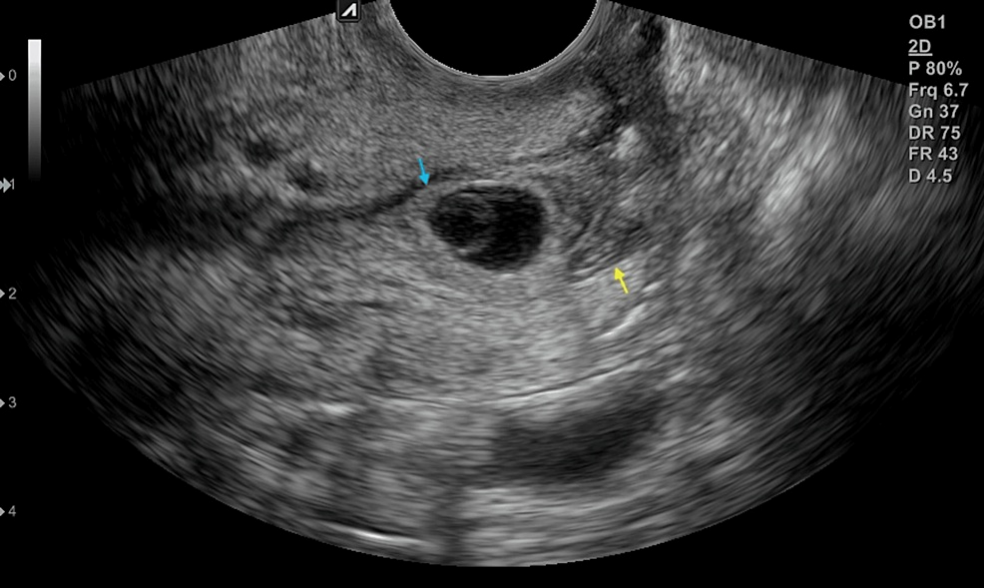
Cervical tissue changes after systemic methotrexate. Decreased echogenicity of the sac rim (light blue arrow) and degeneration of tissues (yellow arrow).

Cervical ectopic pregnancies with poor response to systemic MTX, crown-rump length ≥2.7mm, gestational sac ≤1 mm, and absent cardiac activity may benefit from hydrodissection. The successful outcomes presented in this case could be associated with various factors. Infiltration of lidocaine within the posterolateral regions of the cervix facilitated the displacement and detachment of pregnancy. Outcomes could also be due to prior MTX administration. Although unsuccessful as an initial treatment, MTX could still have affected structures lying proximate to the CEP. In this case, TVUS images after systemic MTX showed decreased echogenicity of the sac’s rim (Figure 3).

A newly developed heterogeneous region, suggestive of tissue degeneration, was also observed within, lying inferior to CEP. DeLoia proposes that MTX derails the development of the trophoblast stem cells [10]. Such a mechanism could have necrotized tissues within the cervical canal, accounting for the changes observed on TVUS (Figure 3). Thus, CEP expulsion could have resulted from the displacement following hydrodissection, yet it was secondary to the weakened attachment and destabilized implantation from prior MTX administration.

## Conclusions

Cervical ectopic pregnancies continue to be rare and life-threatening diagnoses. Although the etiology and pathogenesis remain unknown, risk factors have been identified. These mainly depend on women’s medical, surgical, gynecological, and obstetrical history. As for management, there is currently no standardized approach for CEPs. Methotrexate is the recommended first-line treatment for hemodynamically stable women, administered systemically or locally with US guidance. The success rate of each varies and depends on multiple factors.

Cervical hydrodissection could present favorable assets as a potential alternative for CEPs after failed initial therapy with MTX. Such a combination could reduce the risk of hemorrhage associated with more invasive and traumatic approaches, as well as minimize the risk of incomplete tissue removal following dilation and curettage or manual vacuum aspiration. Nevertheless, further research is vital for determining the underlying mechanisms for expulsion and the role of hydrodissection following MTX as a potential alternative for treatment. Awareness of this event remains vital for future cases.
